# Effects of cortical damage on binocular depth perception

**DOI:** 10.1098/rstb.2015.0254

**Published:** 2016-06-19

**Authors:** Holly Bridge

**Affiliations:** FMRIB Centre, Nuffield Department of Clinical Neurosciences, University of Oxford, John Radcliffe Hospital, Oxford OX3 9DU, UK

**Keywords:** binocular vision, hemianopia, visual system, cortical damage, stereopsis

## Abstract

Stereoscopic depth perception requires considerable neural computation, including the initial correspondence of the two retinal images, comparison across the local regions of the visual field and integration with other cues to depth. The most common cause for loss of stereoscopic vision is amblyopia, in which one eye has failed to form an adequate input to the visual cortex, usually due to strabismus (deviating eye) or anisometropia. However, the significant cortical processing required to produce the percept of depth means that, even when the retinal input is intact from both eyes, brain damage or dysfunction can interfere with stereoscopic vision. In this review, I examine the evidence for impairment of binocular vision and depth perception that can result from insults to the brain, including both discrete damage, temporal lobectomy and more systemic diseases such as posterior cortical atrophy.

This article is part of the themed issue ‘Vision in our three-dimensional world’.

## 1. Introduction

Animals with front facing eyes see the world through two retinal images that are horizontally shifted with respect to each other. The retinal image is only the very first step in a visual pathway that leads through multiple brain areas towards perception. This is particularly the case for depth perception, as monocular information remains segregated until the primary visual cortex (V1) where neurons first receive binocular input.

In V1, there are neurons that have spatially offset receptive fields in the two eyes, which can detect the differences in the retinal images [1–4]. However, a series of experiments have shown that the responses of these V1 neurons do not reflect perceived depth, but rather compute a local correlation between the two images [1,2,5]. For example V1 neurons respond to the disparity in random dot stereograms (RDS) even when the dots are anticorrelated, that is, white dots in one eye are matched to black dots in the other eye, a stimulus that does not lead to perception of depth.

This initial binocular match made in V1 is further processed in V2, with neurons in that area appearing to show a spatial organization for near to far disparities [6], in addition to a specialization for relative disparity processing [7]. Neurophysiological studies in the non-human primate have consistently found that ventral visual areas in the inferotemporal cortex (IT) show neuronal responses corresponding to stereoscopic depth perception. Specifically, neurons in IT respond to correlated, but not anticorrelated, RDS, suggesting that the correspondence problem has been solved [8], and are selective to relative disparity [9,10]. Dorsal areas V3 and V3a, in contrast, appear to be activated by absolute disparity, rather than relative disparity [11].

Unlike the neurophysiological studies, human brain imaging has not highlighted V2 as a region showing specialization for disparity, although this is likely due to the scale of neuronal organization in the area which, at around 1–3 mm, is significantly below the resolution of standard functional magnetic resonance imaging (fMRI; 3 mm voxels). Indeed, human imaging studies have indicated that dorsal regions appear to be the most consistently activated by disparity-defined stimuli [12–20]. However, binocular disparity is only one of several cues to determining depth, and for a full three-dimensional percept it is necessary to integrate other binocular cues (accommodation and vergence) and pictorial cues (e.g. perspective, shading, texture gradients, occlusion) to depth.

The cortical regions responding to depth cues differ according to the specific combination of cues, and the information afforded. Cues related to object perception, such as three-dimensional shape from texture, are represented in the lateral occipital cortex (LOC) while those related to shading include a range of dorsal and ventral regions [21]. Similarly, an earlier study indicated that the lateral regions of visual cortex are involved in processing the combination of disparity and perspective cues [22]. Furthermore, using motion as an additional cue to depth leads to stronger responses in the region around hMT+ and the kinetic occipital area, indicating that these dorsal areas are also involved in the integration of such cues [13].

Thus, there are a number of critical processing stages required to produce a normal stereoscopic depth percept: (i) both eyes aligned and functional^1^; (ii) control over the eye muscles and vergence to bring the images into alignment; (iii) initial matching of retinal images; and (iv) integration of disparity information to produce depth percept. While amblyopia is the most common cause of abnormal binocular vision (see [24] for a recent review), this article covers the much less common deficits in stereoscopic vision resulting from damage to the post-chiasmal visual pathway.

## 2. Effects of cortical damage on binocular vision

**Figure 1.**
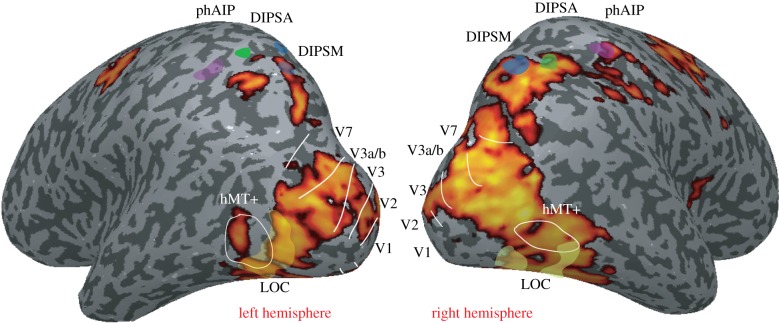
Activation to stimuli defined by binocular disparity. In healthy control subjects, the activation to RDS containing binocular disparity compared to a grey screen with a fixation dot covers a large proportion of the occipital and parietal cortices. White lines indicate the borders of visual areas; transparent yellow regions indicate the position of the regions of interest in the lateral occipital cortex (LOC). Transparent blue region, DIPSM; transparent green, DIPSA; transparent purple, phAIP. Data were *z*-threshold more than 2.3, cluster corrected at *p* = 0.05. Data from Ip *et al*. [16] have been used for this illustration.

The numerous areas of the human visual system activated by binocular disparity and stereoscopic depth are highlighted in figure 1. The widespread activation illustrates the apparent absence of specialized cortical areas to binocular disparity. This observation contrasts with the more localized pattern in which other attributes of the visual world, such as colour or motion, have been found. Area hV4 [25,26] (sometimes referred to as V8 [27]) and additional ventral regions VO1 and VO2 [28] show specificity for chromatic compared to achromatic stimuli. Specialized regions also exist in other visual domains such as hMT+ for motion [29–31], fusiform [32,33] and occipital face areas [34] and LOC for objects [35,36]. Evidence from patients with damage to the occipital cortex provides further support for this functional specialization of visual areas. Damage sustained to the ventral occipital cortex can lead to prosopagnosia [37], achromatopsia [38] or both [39]. Similarly, object agnosia, such as in the case of the much studied patient DF [40,41], can result from damage to LOC, but also damage to ventromedial occipital cortex [42]. There are very few cases of deficits in motion perception, but the most famous case, resulting in static snapshots rather than smooth motion perception, includes the region around motion area hMT+ [43]. While this severe case of akinetopsia was associated with bilateral damage, a few recent reports have indicated that this type of deficit may also arise due to unilateral parietal damage [44,45], or in a more subtle form following unilateral damage to hMT+ [44].

A critical question relates to the type of lesion that might impact upon stereoscopic depth perception in a comparable manner. As the first site of binocular integration, V1 is obviously critical for detecting binocular disparity [1,2,4]. However, damage to this region leads to cortical blindness, so any binocular deficits will not be possible to determine, particularly when the lesion is large. A key test for depth perception is whether the deficit affects local (the matching of individual elements of an image) or global (depth across the whole image) disparity processing. To identify neural structures contributing to stereoscopic depth perception, Cowey & Porter [46] tested the ability of macaque monkeys to detect global stereopsis, rather than local matches, following cortical lesions. They compared performance following lesions to five different occipital regions: V1, V2 and three different regions of the IT. While lesions corresponding to the central region of V1 and V2 had no effect on the animals’ depth performance, the animals with lesions in IT showed impairment of disparity detection. The lack of effect of V1 and V2 lesions is likely due to the relatively small size of the lesions, as much of the stimulus would have been in regions of the visual field unaffected by the lesion. Thus, this experiment suggests that, as might be predicted by the more recent neurophysiological data [8,10], high-level ventral visual regions are necessary for stereoscopic depth perception. Cowey & Wilkinson [47] found that IT damage also raised stereoacuity thresholds for line stimuli presented on the vertical midline. Furthermore, in contrast to the global task, they found that foveal lesions of V2 severely affected the animal's ability to perform this depth task which requires local stereopsis. However, the authors state that they do not know how the animals performed the task, as they were unable to measure fixation or vergence state. Moreover, the extent of other visual deficits in these animals is unknown, so the specificity of this type of lesion to stereoscopic depth perception remains to be determined.

Related to this early non-human primate work, there have been several investigations of the effects on binocular stereopsis of temporal lobectomy performed to relieve intractable epilepsy [48,49]. The most extensive study was that undertaken by Ptito & Zatorre [49] in which, analogous to the work described above, a distinction was made between performance on local and global tests of stereopsis. There was no disruption of performance on the test of local stereopsis in patients with either left- or right-sided lesions compared with healthy control subjects. To determine the level of global stereopsis, the authors used RDS with different levels of correlated dots, from 40 to 100%. All participants (including healthy controls) performed at chance with 40% correlation and all were above 90% correct when the correlation was greater than or equal to 80%. The interesting stimulus range was 50–70% correlation in which the temporal lobectomy patients performed significantly worse than healthy controls. Furthermore, those with right-sided lesions were slightly worse than those with left-sided damage. Thus, it does appear that global stereoscopic depth performance is impaired by damage to the temporal lobe, consistent with findings in the non-human primate. Given these behavioural effects, it would be beneficial to use MRI, both structural and functional, to visualize the exact location of the damage to correlate with these behavioural impairments.

While neurophysiological studies in non-human primates highlight the importance of the ventral visual cortex for stereoscopic depth perception, in human [12,18] (and non-human primate [50,51]) fMRI studies, dorsal regions such as V3a and V7 are consistently activated by disparity-defined depth stimuli (figure 1). Consistent with such a dorsal pathway, one of the earliest reports of a loss of depth perception was in a patient with parietal cortex damage studied by Holmes & Horrax [52]. The report describes difficulty in judging the distance of objects (that the patient often walked into) and a percept of the world as ‘flat’. Subsequent cases in which depth perception has been affected by bilateral damage to the parietal lobes have also been described, although the world is not necessarily described as ‘flat’ [53]. In the case study of Berryhill and colleagues, a patient with bilateral parietal lesions had significant impairment on tests of stereoacuity, but additionally was unable to successfully use monocular cues to depth, such as shape from shading, perspective or size. Thus, this is suggestive of a high-level cue integration impairment, such that it is not possible to integrate the various cues to depth, leaving considerable impairment in visual performance in the depth plane.

A potential explanation of the loss of depth perception leading to a ‘flat’ world is that it is due to a disruption of fusion in the horizontal plane [54]. Schaadt *et al*. presented a patient with an extensive lesion to the right occipito-parietal cortex, who described his world as ‘flat’ such that all objects appeared an equal distance away from him. The patient had normal stereoscopic vision, as assessed by standardized tests (Titmus test and TNO test) but deficits in binocular convergence, i.e. he could not use his two eyes together to focus at a particular depth. Using a training paradigm with prisms designed to improve convergence, the patient reported regaining perception of the third dimension after six sessions, with full-depth perception after 12 sessions. The training did not change the stereoscopic performance, which remained good. It would be interesting to know whether this patient was also impaired in judging depth from monocular cues, as it is of interest to understand whether recovery was due to the unilateral nature of the deficit or whether such training can aid all types of depth perception problems.

A further condition in which there have been several reports of loss of binocular depth perception is traumatic brain injury (TBI). An early report by Hart [55] described a series of patients who had suffered TBI and reported loss of fusion. Of the patients who suffered total loss of fusion, around half showed a full recovery. It is not clear what determined the outcome, but the involvement of the cranial nerves section controlling the extra-ocular muscles (III, IV and VI) is likely to be problematic. A more recent study indicated that a loss of stereopsis resulted from a variety of different types of TBI including both focal parietal damage and more diffuse injury with no obvious focal pathology [56]. However, the correlation between loss of stereoscopic depth perception and measures of recall indicate that cognitive factors may also interfere with this type of stereoscopic testing. If the stereoscopic difficulties following TBI are related to fusion, then it may be that a retraining programme could improve depth perception in such patients. In the study of Schaadt *et al*. [57], fusion training using prismatic and dichoptic devices seemed to improve fusion and binocular stereopsis in around half of the patients with TBI. This suggests that it might be possible to provide improvement in binocular vision in those patients who do not spontaneously regain binocular depth perception following TBI.

In summary, consistent with those areas predicted from the pattern of neural activation to disparity-defined depth, there are regions of both the ventral and dorsal visual streams that can interfere with depth perception when damaged. The extent to which the deficits are due to fusional problems remains to be determined, but this is most likely in the case of involvement of the cranial nerves [55].

## 3. Effects of visual agnosia on binocular vision

There is a general consensus that the primate visual system consists of two parallel, yet highly interconnected, streams: dorsal and ventral. Goodale & Milner [58] suggested that these streams could be considered as a dorsal pathway for action and ventral pathway for perception. The visual behaviour of agnosic patient DF was one of the factors leading to this interpretation, given her ability to use visual information to take action, but inability to recognize objects [40,41]. Since depth information is required in both streams [14,18,59], for object identification and spatial location in space, one could predict that some aspects of stereoscopic depth perception might be affected, while others remain intact.

Given the potential insight that such a lesion could provide, stereoscopic depth performance was studied in great detail by Read *et al*. [60] over several years. They presented a variety of tasks, specifically quantifying DF's ability to use absolute and relative disparity. Indeed, they found that while her performance using absolute disparity was equivalent, if not superior, to naive control participants, her performance did not improve when relative disparity information was available. DF's ability to determine absolute disparity was maintained even when stimulus presentation time was decreased to prevent vergence eye movements, which could aid in this task [61]. Furthermore, patient DF was able to integrate motion and disparity information to determine the direction of rotation of a transparent rotating cylinder. This stimulus is constructed of two planes of dots with sinusoidal velocity profiles in opposite directions. While this is perceived as a rotating cylinder, the direction of rotation is ambiguous, unless a disparity signal of opposite sign is added to the two planes. Previous neurophysiological data have shown that neurons in macaque motion area MT are selective for the direction of rotation [62,63], and stimulation of MT neurons can influence choice of rotation direction [64]. Thus, it seems reasonable that the less impaired dorsal visual stream in DF is able to use binocular disparity in a comparable way to healthy controls.

The psychophysical testing of DF therefore indicated that she could use both absolute disparity and the combination of motion with disparity to determine binocular depth. However, she was considerably impaired at using any type of reference stimulus to improve performance. Whereas those with normal binocular vision can improve their thresholds for detecting stereoscopic depth by the addition of a comparative region, in this form of relative disparity [65,66], she does not appear to show any benefit.

**Figure 2.**
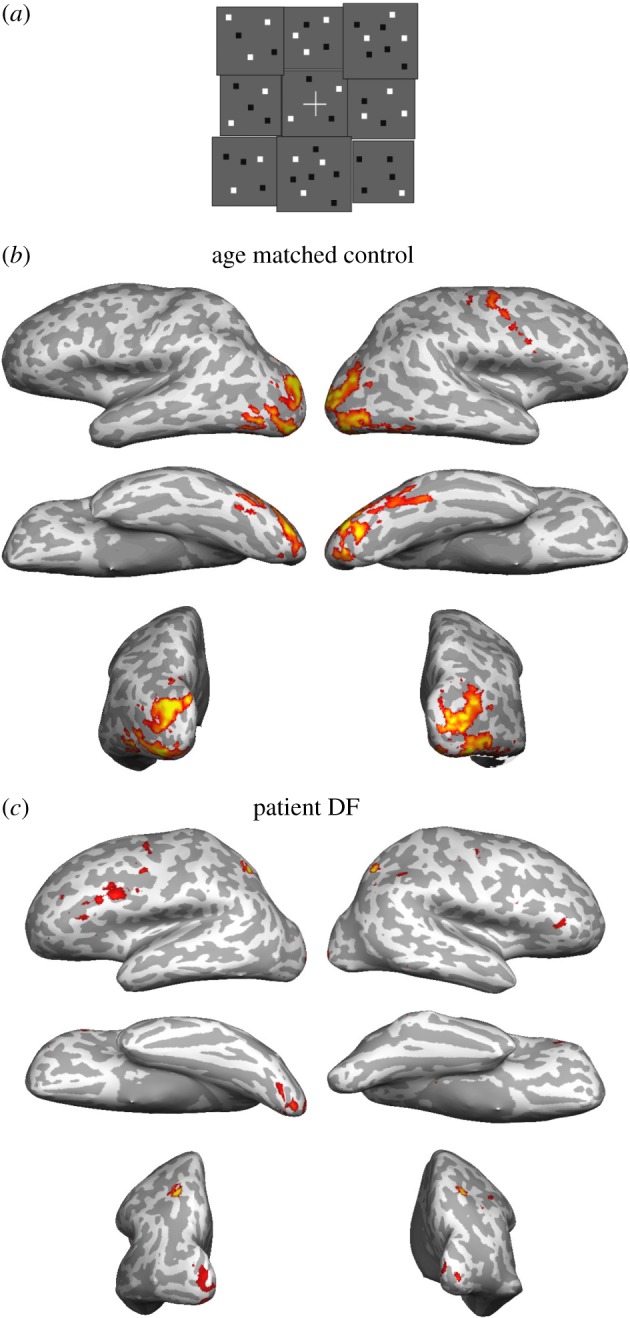
Few cortical areas activate to binocular disparity in patient DF who has visual agnosia. (*a*) A pictorial representation of the disparity-defined checkerboard that was contrasted with a random dot plane at zero disparity. (*b*) The age- and gender-matched control participant showed a standard pattern of activation to a disparity-defined stimulus in the extrastriate regions. (*c*) In spite of DF being able to describe the disparity-defined stimulus shown in (*a*), there was little activation of occipital regions to this stimulus compared with zero-disparity dots. Data from Bridge *et al*. [67].

To determine whether the disparity processing ability of DF was reflected in the neural activation of the occipital cortex, Bridge *et al*. [67] used fMRI during viewing of a disparity-defined checkerboard (figure 2*a*). The disparity of each checkerboard square changed independently to a new value every second during the ‘disparity’ condition and was contrasted to a zero-disparity plane. Figure 2*b* shows the activation in an example control subject and, similar to the activity in figure 1, this resulted in activation across considerable regions of the occipital lobe. Interestingly, in patient DF, despite her relatively good disparity performance and the fact that she could describe the stimulus, there is very little neural activity. The activity is confined to a small region of the ventral occipital lobe. It is not clear why the level of activity should be so low; the activity to a moving stimulus was considerably higher than that to disparity-defined stimuli, suggesting a general loss of neural activity or vasculature was not the cause. However, it may be that the network for detecting binocular disparity is reduced in activity level, but still able to contribute to perception.

While agnosic patient DF has been tested extensively on depth perception from stereoscopic information, there is little data to determine the effects of other, pictorial cues, on her overall depth perception. Interestingly, another agnosic patient, DM, showed considerable impairment on extracting depth information from pictorial cues [68]. In particular, the patient was unable to extract three-dimensional structure from line drawings to discriminate ‘possible’ and ‘impossible’ objects or to perform three-dimensional object rotation. Unfortunately, stereoscopic depth perception was not tested in this patient, so it is not possible to compare the findings directly to DF.

## 4. The contribution of the corpus callosum to stereoscopic depth perception

**Figure 3.**
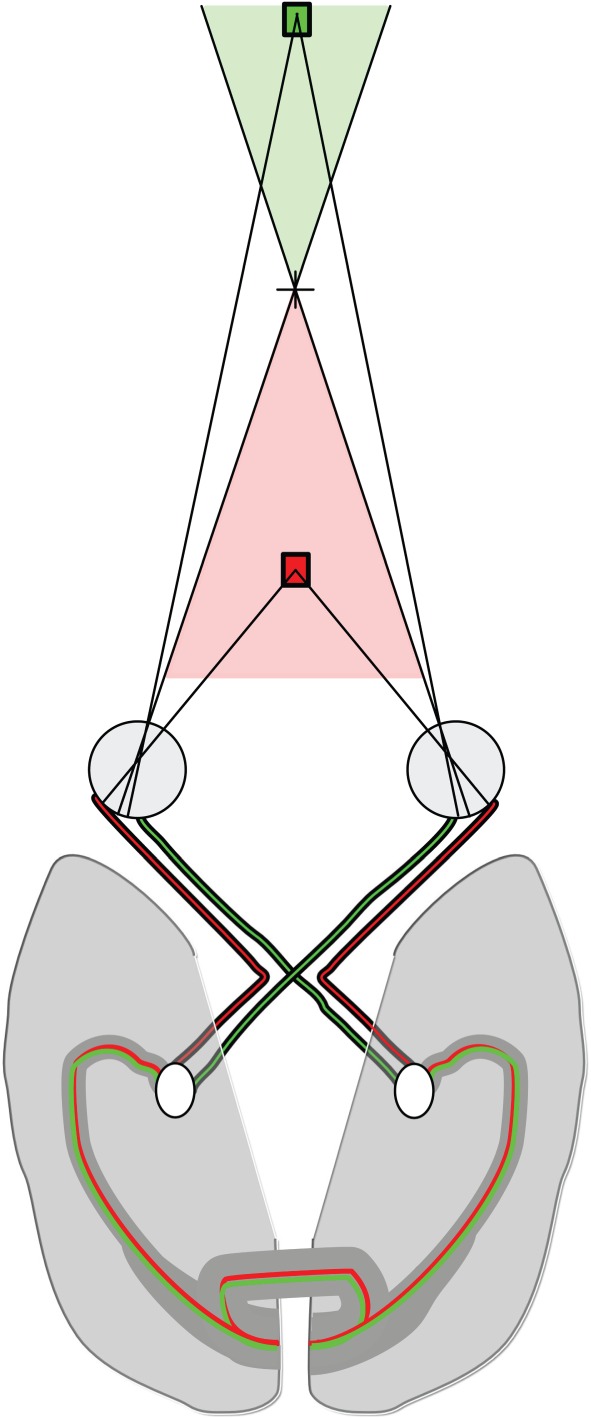
The role of the corpus callosum in binocular stereopsis. The images of objects on the vertical meridian fall onto parts of the retina that project to different hemispheres. Near, crossed disparities (red region) fall onto the temporal retinas and far, uncrossed disparities (green region) fall onto the nasal retinas. Thus, to compare the stimuli falling in these regions, information needs to be compared across hemispheres, using the corpus callosum.

Binocular stereopsis, as laid out in most of this article, is based predominantly on the direct projections from the lateral geniculate nucleus (LGN) of the thalamus, where the information from the two eyes remains separate. By combining these inputs on binocular neurons with slightly spatially offset receptive fields or with a shift in receptive field shape [69–71], an initial calculation of absolute disparity can be made. However, when the object containing disparity is located on the vertical meridian, the images of the object fall onto either the nasal retina of both eyes (far disparities) or the temporal retina of both eyes (near disparities). Thus, the images are projected to opposite hemispheres, as shown in figure 3. In this case, communication between the hemispheres is required to determine the corresponding points in the two images. Indeed, Berlucchi & Rizzolatti [72] showed that following section of the optic chiasm in cat (thus eliminating the binocular overlap of the visual fields) a small minority of V1 neurons could be activated by stimuli presented to the contralateral, as well as the ipsilateral, eye. The monocular receptive fields lay close to the vertical meridian, in comparable locations, suggesting that these binocular receptive fields could also detect binocular disparities.

If the corpus callosum is absent or split, it should be possible to identify deficits of midline disparities, with preservation of stereoscopic depth perception across the remainder of the visual field. An early case study by Blakemore & Mitchell [73] investigated performance in tests of binocular stereopsis in a young patient who had undergone surgical callosal section. Although this participant appeared to be unable to determine the relative depth of slit stimuli containing disparity when they were presented to the central visual field, but not peripherally, only a few trials were undertaken and there were no control data. Later work by Jeeves [74] investigated stereoscopic depth performance in four patients, two of whom had the congenital absence of the corpus callosum and two had partial callosal sections. All were compared to control subjects with normal binocular vision. The patients with complete section showed deficits in stereoscopic depth perception tasks at the midline, but not in the periphery. Similarly, the single patient with partial section affecting the splenium also showed deficits that were not present in the patient with anterior section of the corpus callosum. In these three patients, stimuli were rarely perceived as behind fixation, suggesting that even distinguishing near and far disparities was challenging.

The macaque study of Cowey & Wilkinson [47] described earlier also investigated the effects of splenium section on their stereoacuity task. Interestingly, they found no increase in threshold even though the task was presented on the vertical midline. However, without measurements of vergence and fixation, it is not possible to know exactly where the images of the stimulus fell on the retina.

Thus, the early human literature suggested a role for the corpus callosum on midline stereoscopic depth perception, while animal experiments have been inconclusive. A more recent study [75] used a dichoptic plaid stimulus that required binocular integration in order to correctly perceive drift direction. The tasks were performed by two acallosal patients and a healthy control group, and monocular and binocular presentation of the stimulus were used as control tasks. While the patients lacking the corpus callosum correctly perceived locations in depth away from the vertical meridian, performance with the dichoptic viewing (requiring binocular integration) was severely impaired on the vertical midline. When stimulus size was increased, the patients improved their performance, presumably as the stimulus could then be processed by binocular neurons in the contralateral hemisphere alone. The visual evoked potentials recorded from the patients also showed abnormal patterns when the small, dichoptic stimuli were presented, consistent with the perceptual deficit.

## 5. Depth perception in bitemporal hemianopia

**Figure 4.**
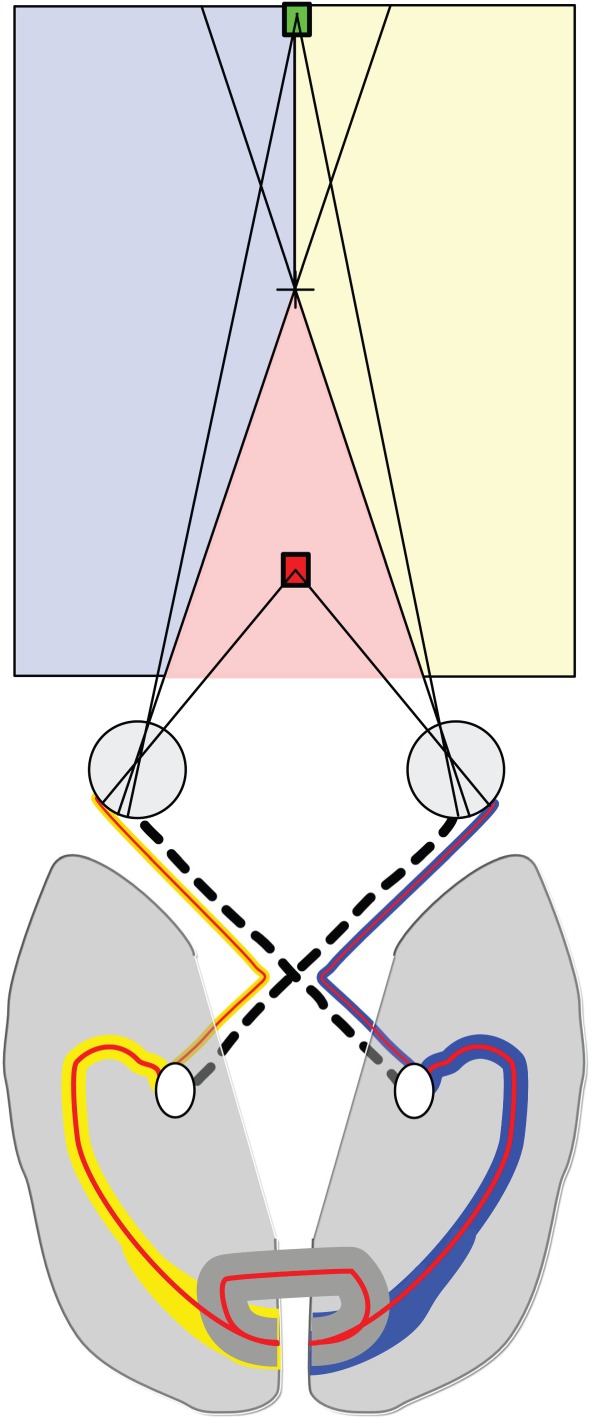
In bitemporal hemianopia, the nasal input to the cortex is lost, meaning that each hemisphere only receives input from one of the eyes (the left hemisphere, yellow, only receives input from the right visual field). Thus, there are no areas of binocular overlap. However, since binocular input on the midline is provided via the corpus callosum, the red region indicates an area in which binocular combination is possible.

Homonymous hemianopia is a loss of vision in one hemifield, usually due to damage to V1, but also the optic radiation or LGN. By contrast, bitemporal hemianopia arises from damage to the optic chiasm, such that the nasal fibres taking information from the temporal retinae are damaged. While the loss of visual field in this condition is relatively small, the outcome is that V1 in each hemisphere only receives information from a single eye (the ipsilateral one), as shown in figure 4. Therefore, the only potential source of binocular integration is across the corpus callosum as described in the previous section. However, even using this information, any images projecting to the nasal retinas will be lost as the nasal projections are severed at the chiasm. Therefore, the only region in which there is potential binocular input is the crossed **‘**near’ disparities along the midline that project to the nasal portion of each eye.

Ablation of the chiasm in cats causes profound difficulties in discrimination of depth in RDS [76]. Similarly, there have been a number of studies investigating patients who have either transient or permanent malfunction of the optic chiasm. In cases where the optic chiasm is compressed due to a tumour, such as a pituitary adenoma, stereoscopic depth perception can be severely disrupted [77]. However, in many cases, surgery to relieve the pressure led to restoration of binocular function. Where the damage is permanent, however, patients are left with very little binocular function. There are very few studies in which stereoscopic depth perception has been systematically studied, although a recent study investigated the deficits in two patients with bitemporal hemianopia [78]. Under normal binocular viewing, mismatched images in the two eyes can stimulate fusional eye movements to align the images [79]. However, in bitemporal hemianopia, the two eyes see different regions of the visual field, so patients can have difficulty establishing fusion. Any eye misalignment will cause double vision if the images overlap (in the case of esotropia) or a vertical scotoma in the case of exotropia. To help the two patients who reported double vision, Peli and Satgunam exploited the intact midline stereopsis (via the corpus callosum) to design a stereo-typoscope that could be used for reading.

A final word on depth perception related to the vertical meridian is the consequences of complete homonymous hemianopia on depth perception in this region. In this case, V1 is damaged on one side, while the unaffected hemisphere will receive binocular information from the contralateral visual field, allowing for stereoscopic depth perception. However, in the absence of macular sparing, one would predict a deficit in computing ‘near’ and ‘far’ binocular disparities on the midline as there are no neurons to communicate via the corpus callosum. While this phenomenon was investigated almost a century ago, and gave a hint that patients with hemianopia did show abnormal stereoscopic performance close to fixation [80], it remains largely unexplored and would be an interesting question to explore with modern psychophysical and imaging methods.

## 6. Depth perception in posterior cortical atrophy and Alzheimer's disease

While the typical presentation of Alzheimer's disease (AD) is memory loss and personality changes, atypical AD, also known as posterior cortical atrophy (PCA) can present with visual difficulties, such as reading, judging distances and identifying objects [81]. As with the typical presentation, PCA is a neurodegenerative condition, such that the initial visuospatial dysfunction can eventually progress to affect the medial temporal lobe primarily affected in AD. There have been suggestions of different PCA subtypes that might differentially affect either the dorsal or ventral visual systems, or even the striate cortex [82,83]. Thus, the effects on the stereoscopic depth system are difficult to predict. A recent study [84] compared performance on psychophysical tests of depth perception in patients with PCA and typical AD. They also included patients with diffuse Lewy body dementia (DLBD), because it is known to lead to disruption of visual perception [85]. In particular, this study aimed to investigate the specific effects of the diseases on different types of depth cue: three-dimensional shape from disparity, texture, shading or motion. Interestingly, the PCA group showed no deficit compared with healthy controls in perceiving three-dimensional shape from disparity, but the three monocular cue conditions were all relatively impaired. However, these patients did not differ from the DLBD patients for any stimulus, and were worse than AD patients only in the shape from texture condition. Although the PCA patients were also impaired at discriminating the basic features of the stimuli, the level of impairment was not sufficient to account for the three-dimensional shape deficit.

There was considerable variability in the performance of the PCA patients within the group, which might reflect the different pattern of damage that has been seen in previous studies. To take account of this variability, the authors correlated the performance on each of the three-dimensional tasks with grey matter volume across the group. Interestingly, these correlation analyses identified distinct ventral occipital regions for two of the three-dimensional tasks. The shape from disparity performance correlated with grey matter loss in a region of right inferior temporal cortex; the region correlating with performance in shape from disparity was more posterior and superior. The neural response to depth from disparity in healthy control subjects, shown in figure 1, includes significant activation in this damaged area, also seen in other studies [14,16–20].

Investigating stereoscopic depth perception in dementia is particularly challenging since the patients need to understand the nature of the task. Thus, rather than just being compared to healthy older participants, non-depth visual tasks are required to establish that there is not a general visual or comprehension deficit. Thus, some early studies found AD patients to be impaired on stereoscopic depth perception [86] but did not fully control for a general decline in visual function. A more recent study asked patients with AD and Parkinson's disease to rate a three-dimensional movie on how good the depth looked, but this type of approach is likely to be challenging for patients experiencing memory problems [87]. By contrast, the study described earlier [84] used typical AD patients as a control group and concluded that, although there was some impairment of extracting three-dimensional shape, this was likely as a result of downstream processes, potentially at the level of decision-making.

## 7. Concluding remarks

The most common cause of dysfunctional stereoscopic depth perception is amblyopia, generally affecting visual regions at the level of V1 and the anterior visual pathway. However, while this type of dysfunction occurs at the level of integration of binocular information, damage to higher cortical visual areas can also interfere with depth perception. While training in fusional techniques may improve some aspects of depth perception, these techniques are unlikely to account for all cases, particularly those in which the deficit appears to be at the level of integration of monocular and binocular cues to depth.

## Endnotes

1 Note that it is possible to produce a stereoscopic depth percept without correct alignment of the eyes, known as Anomalous Retinal Correspondence. The binocular information in this case, however, is the most informative when the misalignment is small [23].

## References

[1] Cumming BG, Parker AJ 1997 Responses of primary visual cortical neurons to binocular disparity without depth perception. Nature 389, 280–283. (10.1038/38487) 9305841

[2] Cumming BG, Parker AJ 1999 Binocular neurons in V1 of awake monkeys are selective for absolute, not relative, disparity. J. Neurosci. 19, 5602–5618. 10377367 10.1523/JNEUROSCI.19-13-05602.1999 PMC6782336

[3] DeAngelis GC, Ohzawa I, Freeman RD 1995 Neuronal mechanisms underlying stereopsis: how do simple cells in the visual cortex encode binocular disparity? Perception 24, 3–31. (10.1068/p240003) 7617416

[4] Barlow HB, Blakemore C, Pettigrew JD 1967 The neural mechanism of binocular depth discrimination. J. Physiol. 193, 327–342. (10.1113/jphysiol.1967.sp008360) 6065881 PMC1365600

[5] Cumming BG, Parker AJ 2000 Local disparity not perceived depth is signaled by binocular neurons in cortical area V1 of the macaque. J. Neurosci. 20, 4758–4767. 10844045 10.1523/JNEUROSCI.20-12-04758.2000 PMC6772469

[6] Chen G, Lu HD, Roe AW 2008 A map for horizontal disparity in monkey V2. Neuron 58, 442–450. (10.1016/j.neuron.2008.02.032) 18466753 PMC2441920

[7] Thomas OM, Cumming BG, Parker AJ 2002 A specialization for relative disparity in V2. Nat. Neurosci. 5, 472–478. (10.1038/nn837) 11967544

[8] Janssen P, Vogels R, Liu Y, Orban GA 2003 At least at the level of inferior temporal cortex, the stereo correspondence problem is solved. Neuron 37, 693–701. (10.1016/S0896-6273(03)00023-0) 12597865

[9] Orban GA, Janssen P, Vogels R. 2006 Extracting 3D structure from disparity. Trends Neurosci. 29, 466–473. (10.1016/j.tins.2006.06.012) 16842865

[10] Janssen P, Vogels R, Orban GA 1999 Macaque inferior temporal neurons are selective for disparity-defined three-dimensional shapes. Proc. Natl Acad. Sci. USA 96, 8217–8222. (10.1073/pnas.96.14.8217) 10393975 PMC22215

[11] Anzai A, Chowdhury SA, DeAngelis GC 2011 Coding of stereoscopic depth information in visual areas V3 and V3A. J. Neurosci. 31, 10 270–10 282. (10.1523/JNEUROSCI.5956-10.2011) PMC3143190 21753004

[12] Backus BT, Fleet DJ, Parker AJ, Heeger DJ 2001 Human cortical activity correlates with stereoscopic depth perception. J. Neurophysiol. 86, 2054–2068. 11600661 10.1152/jn.2001.86.4.2054

[13] Ban H, Preston TJ, Meeson A, Welchman AE 2012 The integration of motion and disparity cues to depth in dorsal visual cortex. Nat. Neurosci. 15, 636–643. (10.1038/nn.3046) 22327475 PMC3378632

[14] Bridge H, Parker AJ 2007 Topographical representation of binocular depth in the human visual cortex using fMRI. J. Vis. 7, 15 (10.1167/7.14.15) 18217810

[15] Georgieva S, Peeters R, Kolster H, Todd JT, Orban GA 2009 The processing of three-dimensional shape from disparity in the human brain. J. Neurosci. 29, 727–742. (10.1523/JNEUROSCI.4753-08.2009) 19158299 PMC6665151

[16] Ip IB, Minini L, Dow J, Parker AJ, Bridge H. 2014 Responses to interocular disparity correlation in the human cerebral cortex. Ophthalmic Physiol. Opt. 34, 186–198. (10.1111/opo.12121) 24588533 PMC4265194

[17] Minini L, Parker AJ, Bridge H. 2010 Neural modulation by binocular disparity greatest in human dorsal visual stream. J. Neurophysiol. 104, 169–178. (10.1152/jn.00790.2009) 20445027 PMC2904223

[18] Neri P, Bridge H, Heeger DJ 2004 Stereoscopic processing of absolute and relative disparity in human visual cortex. J. Neurophysiol. 92, 1880–1891. (10.1152/jn.01042.2003) 15331652

[19] Preston TJ, Li S, Kourtzi Z, Welchman AE 2008 Multivoxel pattern selectivity for perceptually relevant binocular disparities in the human brain. J. Neurosci. 28, 11 315–11 327. (10.1523/JNEUROSCI.2728-08.2008) PMC6671500 18971473

[20] Tsao DY, Conway BR, Livingstone MS 2003 Receptive fields of disparity-tuned simple cells in macaque V1. Neuron 38, 103–114. (10.1016/S0896-6273(03)00150-8) 12691668 PMC8143702

[21] Georgieva SS, Todd JT, Peeters R, Orban GA 2008 The extraction of 3D shape from texture and shading in the human brain. Cereb. Cortex 18, 2416–2438. (10.1093/cercor/bhn002) 18281304 PMC2536698

[22] Welchman AE, Deubelius A, Conrad V, Bulthoff HH, Kourtzi Z. 2005 3D shape perception from combined depth cues in human visual cortex. Nat. Neurosci. 8, 820–827. (10.1038/nn1461) 15864303

[23] Herzau V 1996 How useful is anomalous correspondence? Eye 10, 266–269. (10.1038/eye.1996.56) 8776458

[24] Levi DM, Knill DC, Bavelier D. 2015 Stereopsis and amblyopia: a mini-review. Vis. Res. 114, 17–30. (10.1016/j.visres.2015.01.002) 25637854 PMC4519435

[25] Wade A, Augath M, Logothetis N, Wandell B 2008 fMRI measurements of color in macaque and human. J. Vis. 8, 6 (10.1167/8.10.6) PMC3045694 19146348

[26] Wade AR, Brewer AA, Rieger JW, Wandell BA 2002 Functional measurements of human ventral occipital cortex: retinotopy and colour. Phil. Trans. R. Soc. Lond. B 357, 963–973. (10.1098/rstb.2002.1108) 12217168 PMC1693014

[27] Hadjikhani N, Liu AK, Dale AM, Cavanagh P, Tootell RB 1998 Retinotopy and color sensitivity in human visual cortical area V8. Nat. Neurosci. 1, 235–241. (10.1038/681) 10195149

[28] Wandell BA, Winawer J. 2011 Imaging retinotopic maps in the human brain. Vis. Res. 51, 718–737. (10.1016/j.visres.2010.08.004) 20692278 PMC3030662

[29] Watson JD, Myers R, Frackowiak RS, Hajnal JV, Woods RP, Mazziotta JC, Shipp S, Zeki S 1993 Area V5 of the human brain: evidence from a combined study using positron emission tomography and magnetic resonance imaging. Cereb. Cortex 3, 79–94. (10.1093/cercor/3.2.79) 8490322

[30] Huk AC, Dougherty RF, Heeger DJ 2002 Retinotopy and functional subdivision of human areas MT and MST. J. Neurosci. 22, 7195–7205. 12177214 10.1523/JNEUROSCI.22-16-07195.2002 PMC6757870

[31] Amano K, Wandell BA, Dumoulin SO 2009 Visual field maps, population receptive field sizes, and visual field coverage in the human MT+ complex. J. Neurophysiol. 102, 2704–2718. (10.1152/jn.00102.2009) 19587323 PMC2777836

[32] Grill-Spector K, Knouf N, Kanwisher N. 2004 The fusiform face area subserves face perception, not generic within-category identification. Nat. Neurosci. 7, 555–562. (10.1038/nn1224) 15077112

[33] Kanwisher N, McDermott J, Chun MM 1997 The fusiform face area: a module in human extrastriate cortex specialized for face perception. J. Neurosci. 17, 4302–4311. 9151747 10.1523/JNEUROSCI.17-11-04302.1997 PMC6573547

[34] Rossion B, Caldara R, Seghier M, Schuller AM, Lazeyras F, Mayer E. 2003 A network of occipito-temporal face-sensitive areas besides the right middle fusiform gyrus is necessary for normal face processing. Brain 126, 2381–2395. (10.1093/brain/awg241) 12876150

[35] Kourtzi Z, Kanwisher N 2001 Representation of perceived object shape by the human lateral occipital complex. Science 293, 1506–1509. (10.1126/science.1061133) 11520991

[36] Grill-Spector K, Kourtzi Z, Kanwisher N 2001 The lateral occipital complex and its role in object recognition. Vis. Res. 41, 1409–1422. (10.1016/S0042-6989(01)00073-6) 11322983

[37] Hadjikhani N, de Gelder B. 2002 Neural basis of prosopagnosia: an fMRI study. Hum. Brain Mapping 16, 176–182. (10.1002/hbm.10043) PMC6871884 12112771

[38] Damasio A, Yamada T, Damasio H, Corbett J, McKee J 1980 Central achromatopsia: behavioral, anatomic, and physiologic aspects. Neurology 30, 1064–1071. (10.1212/WNL.30.10.1064) 6968419

[39] Bouvier SE, Engel SA 2006 Behavioral deficits and cortical damage loci in cerebral achromatopsia. Cereb. Cortex 16, 183–191. (10.1093/cercor/bhi096) 15858161

[40] James TW, Culham J, Humphrey GK, Milner AD, Goodale MA 2003 Ventral occipital lesions impair object recognition but not object-directed grasping: an fMRI study. Brain 126, 2463–2475. (10.1093/brain/awg248) 14506065

[41] Milner AD et al 1991 Perception and action in ‘visual form agnosia’. Brain 114, 405–428. (10.1093/brain/114.1.405) 2004249

[42] Karnath HO, Ruter J, Mandler A, Himmelbach M. 2009 The anatomy of object recognition--visual form agnosia caused by medial occipitotemporal stroke. J. Neurosci. 29, 5854–5862. (10.1523/JNEUROSCI.5192-08.2009) 19420252 PMC6665227

[43] Zihl J, von Cramon D, Mai N 1983 Selective disturbance of movement vision after bilateral brain damage. Brain 106, 313–340. (10.1093/brain/106.2.313) 6850272

[44] Cooper SA, Joshi AC, Seenan PJ, Hadley DM, Muir KW, Leigh RJ, Metcalfe RA 2012 Akinetopsia: acute presentation and evidence for persisting defects in motion vision. J. Neurol. Neurosurg. Psychiatry 83, 229–230. (10.1136/jnnp.2010.223727) 21217160

[45] Sakurai K, Kurita T, Takeda Y, Shiraishi H, Kusumi I. 2013 Akinetopsia as epileptic seizure. Epilepsy Behav. 1, 74–76. (10.1016/j.ebcr.2013.04.002) PMC4150625 25667833

[46] Cowey A, Porter J 1979 Brain damage and global stereopsis. Proc. R. Soc. Lond. B 204, 399–407. (10.1098/rspb.1979.0035) 38454

[47] Cowey A, Wilkinson F 1991 The role of the corpus callosum and extra striate visual areas in stereoacuity in macaque monkeys. Neuropsychologia 29, 465–479. (10.1016/0028-3932(91)90005-S) 1944856

[48] Ptito A, Zatorre RJ 1988 Impaired stereoscopic detection thresholds after left or right temporal lobectomy. Neuropsychologia 26, 547–554. (10.1016/0028-3932(88)90111-X) 3405400

[49] Ptito A, Zatorre RJ, Larson WL, Tosoni C 1991 Stereopsis after unilateral anterior temporal lobectomy. Dissociation between local and global measures. Brain 114, 1323–1333. (10.1093/brain/114.3.1323) 2065253

[50] Tsao DY et al. 2003 Stereopsis activates V3A and caudal intraparietal areas in macaques and humans. Neuron 39, 555–568. (10.1016/S0896-6273(03)00459-8) 12895427

[51] Verhoef BE, Bohon KS, Conway BR 2015 Functional architecture for disparity in macaque inferior temporal cortex and its relationship to the architecture for faces, color, scenes, and visual field. J. Neurosci. 35, 6952–6968. (10.1523/JNEUROSCI.5079-14.2015) 25926470 PMC4412905

[52] Holmes G, Horrax G 1919 Disturbances of spatial orientation and visual attention, with loss of stereoscopic vision. Arch. Neurol. Psychiatry 1, 384–407. (10.1001/archneurpsyc.1919.02180040002001)

[53] Berryhill ME, Fendrich R, Olson IR 2009 Impaired distance perception and size constancy following bilateral occipitoparietal damage. Exp. Brain Res. 194, 381–393. (10.1007/s00221-009-1707-7) 19183969 PMC2728930

[54] Schaadt AK, Brandt SA, Kraft A, Kerkhoff G. 2015 Holmes and Horrax (1919) revisited: impaired binocular fusion as a cause of ‘flat vision’ after right parietal brain damage: a case study. Neuropsychologia 69, 31–38. (10.1016/j.neuropsychologia.2015.01.029) 25619849

[55] Hart CT 1969 Disturbances of fusion following head injury. Proc. R. Soc. Med. 62, 704–706. 5803516 10.1177/003591576906200731 PMC1815512

[56] Miller LJ, Mittenberg W, Carey VM, McMorrow MA, Kushner TE, Weinstein JM 1999 Astereopsis caused by traumatic brain injury. Arch. Clin. Neuropsychol. 14, 537–543. (10.1093/arclin/14.6.537) 14590581

[57] Schaadt AK, Schmidt L, Kuhn C, Summ M, Adams M, Garbacenkaite R, Leonhardt E, Reinhart S, Kerkhoff G. 2014 Perceptual relearning of binocular fusion after hypoxic brain damage: four controlled single-case treatment studies. Neuropsychology 28, 382–387. (10.1037/neu0000019) 24188115

[58] Goodale MA, Milner AD 1992 Separate visual pathways for perception and action. Trends Neurosci. 15, 20–25. (10.1016/0166-2236(92)90344-8) 1374953

[59] Parker AJ 2007 Binocular depth perception and the cerebral cortex. Nat. Rev. Neurosci. 8, 379–391. (10.1038/nrn2131) 17453018

[60] Read JC, Phillipson GP, Serrano-Pedraza I, Milner AD, Parker AJ 2010 Stereoscopic vision in the absence of the lateral occipital cortex. PLoS ONE 5, e12608 (10.1371/journal.pone.0012608) 20830303 PMC2935377

[61] Mon-Williams M, Tresilian JR, McIntosh RD, Milner AD 2001 Monocular and binocular distance cues: insights from visual form agnosia I (of III). Exp. Brain Res. 139, 127–136. (10.1007/s002210000658) 11497053

[62] Krug K, Cumming BG, Parker AJ 2004 Comparing perceptual signals of single V5/MT neurons in two binocular depth tasks. J. Neurophysiol. 92, 1586–1596. (10.1152/jn.00851.2003) 15102899

[63] Dodd JV, Krug K, Cumming BG, Parker AJ 2001 Perceptually bistable three-dimensional figures evoke high choice probabilities in cortical area MT. J. Neurosci. 21, 4809–4821. 11425908 10.1523/JNEUROSCI.21-13-04809.2001 PMC6762355

[64] Krug K, Cicmil N, Parker AJ, Cumming BG 2013 A causal role for V5/MT neurons coding motion-disparity conjunctions in resolving perceptual ambiguity. Curr. Biol. 23, 1454–1459. (10.1016/j.cub.2013.06.023) 23871244 PMC3739008

[65] Andrews TJ, Glennerster A, Parker AJ 2001 Stereoacuity thresholds in the presence of a reference surface. Vis. Res. 41, 3051–3061. (10.1016/S0042-6989(01)00192-4) 11704242

[66] Westheimer G 1979 Cooperative neural processes involved in stereoscopic acuity. Exp. Brain Res. 36, 585–597. (10.1007/BF00238525) 477784

[67] Bridge H, Thomas OM, Minini L, Cavina-Pratesi C, Milner AD, Parker AJ 2013 Structural and functional changes across the visual cortex of a patient with visual form agnosia. J. Neurosci. 33, 12 779–12 791. (10.1523/JNEUROSCI.4853-12.2013) PMC6618540 23904613

[68] Turnbull OH, Driver J, McCarthy RA 2004 2D but not 3D: pictorial-depth deficits in a case of visual agnosia. Cortex 40, 723–738. (10.1016/S0010-9452(08)70167-9) 15505981

[69] Prince SJ, Cumming BG, Parker AJ 2002 Range and mechanism of encoding of horizontal disparity in macaque V1. J. Neurophysiol. 87, 209–221. 11784743 10.1152/jn.00466.2000

[70] Prince SJ, Pointon AD, Cumming BG, Parker AJ 2002 Quantitative analysis of the responses of V1 neurons to horizontal disparity in dynamic random-dot stereograms. J. Neurophysiol. 87, 191–208. 11784742 10.1152/jn.00465.2000

[71] Ohzawa I, DeAngelis GC, Freeman RD 1990 Stereoscopic depth discrimination in the visual cortex: neurons ideally suited as disparity detectors. Science 249, 1037–1041. (10.1126/science.2396096) 2396096

[72] Berlucchi G, Rizzolatti G 1968 Binocularly driven neurons in visual cortex of split-chiasm cats. Science 159, 308–310. (10.1126/science.159.3812.308) 5634497

[73] Mitchell DE, Blakemore C 1970 Binocular depth perception and the corpus callosum. Vis. Res. 10, 49–54. (10.1016/0042-6989(70)90061-1) 5435012

[74] Jeeves MA 1991 Stereo perception in callosal agenesis and partial callosotomy. Neuropsychologia 29, 19–34. (10.1016/0028-3932(91)90091-L) 2017306

[75] Saint-Amour D, Lepore F, Lassonde M, Guillemot JP 2004 Effective binocular integration at the midline requires the corpus callosum. Neuropsychologia 42, 164–174. (10.1016/j.neuropsychologia.2003.07.002) 14644103

[76] Lepore F, Ptito M, Lassonde M 1986 Stereoperception in cats following section of the corpus callosum and/or the optic chiasma. Exp. Brain Res. 61, 258–264. (10.1007/BF00239515) 3948940

[77] Hirai T, Ito Y, Arai M, Ota Y, Kojima T, Sato M, Miyake Y 2002 Loss of stereopsis with optic chiasmal lesions and stereoscopic tests as a differential test. Ophthalmology 109, 1692–1702. (10.1016/S0161-6420(02)01171-5) 12208719

[78] Peli E, Satgunam P. 2014 Bitemporal hemianopia; its unique binocular complexities and a novel remedy. Ophthalmic Physiol. Opt. 34, 233–242. (10.1111/opo.12118) 24588535 PMC3947624

[79] Masson GS, Busettini C, Miles FA 1997 Vergence eye movements in response to binocular disparity without depth perception. Nature 389, 283–286. (10.1038/38496) 9305842

[80] Brown EV, Kronfeld PC 1930 The acuity of binocular depth perception in hemianopsia. Trans. Am. Ophthalmol. Soc. 28, 231–249. 16692862 PMC1316771

[81] Crutch SJ, Lehmann M, Schott JM, Rabinovici GD, Rossor MN, Fox NC 2012 Posterior cortical atrophy. Lancet Neurol. 11, 170–178. (10.1016/S1474-4422(11)70289-7) 22265212 PMC3740271

[82] Ross SJ, Graham N, Stuart-Green L, Prins M, Xuereb J, Patterson K, Hodges JR 1996 Progressive biparietal atrophy: an atypical presentation of Alzheimer's disease. J. Neurol. Neurosurg. Psychiatry 61, 388–395. (10.1136/jnnp.61.4.388) 8890778 PMC486580

[83] Galton CJ, Patterson K, Xuereb JH, Hodges JR 2000 Atypical and typical presentations of Alzheimer's disease: a clinical, neuropsychological, neuroimaging and pathological study of 13 cases. Brain 123, 484–498. (10.1093/brain/123.3.484) 10686172

[84] Gillebert CR et al. 2015 3D shape perception in posterior cortical atrophy: a visual neuroscience perspective. J. Neurosci. 35, 12 673–12 692. (10.1523/JNEUROSCI.3651-14.2015) PMC4571603 26377458

[85] Mori E, Shimomura T, Fujimori M, Hirono N, Imamura T, Hashimoto M, Tanimukai S, Kazui H, Hanihara T 2000 Visuoperceptual impairment in dementia with Lewy bodies. Arch. Neurol. 57, 489–493. (10.1001/archneur.57.4.489) 10768622

[86] Mittenberg W, Malloy M, Petrick J, Knee K 1994 Impaired depth perception discriminates Alzheimer's dementia from aging and major depression. Arch. Clin. Neuropsychol. 9, 71–79. (10.1093/arclin/9.1.71) 14589513

[87] Lee CN, Ko D, Suh YW, Park KW 2015 Cognitive functions and stereopsis in patients with Parkinson's disease and Alzheimer's disease using 3-dimensional television: a case controlled trial. PLoS ONE 10, e0123229 (10.1371/journal.pone.0123229) 25822839 PMC4378891

